# Hiccup: The Striking Manifestation of Hyponatremia Due to Ischemic Stroke-Induced Cerebral Salt Wasting Syndrome (CSWS)

**DOI:** 10.7759/cureus.29677

**Published:** 2022-09-28

**Authors:** Utsow Saha, Anid Hassan, Chowdhury F Zaman, Sabrina Afroz, Ridwan Faruq

**Affiliations:** 1 Medical Education, Enam Medical College and Hospital, Rangpur, BGD; 2 Department of Internal Medicine, Holy Family Red Crescent Medical College Hospital, Dhaka, BGD; 3 Medicine and Surgery, Jahurul Islam Medical College and Hospital, Kishoregonj, BGD; 4 Internal Medicine, Tri-City Medical Group, Los Angeles, USA; 5 Internal Medicine, Yale New Haven Bridgeport Hospital, Bridgeport, USA

**Keywords:** syndrome of inappropriate antidiuresis, cerebral salt-wasting syndrome, cerebro-vascular accident (stroke), hyponatremia, hiccup

## Abstract

Among many clinical symptoms, hiccups are an infrequent presentation of hyponatremia. Hyponatremia indicates a serum sodium level of less than 135 mmol/l, the most common reported electrolyte abnormality. Cerebral salt wasting syndrome is a less common cause of hyponatremia, which can arise from a spectrum of brain pathology. This case report brings attention to a case of hyponatremia due to cerebral salt wasting syndrome in a 76-year-old man who suffered from an ischemic stroke. The hyponatremia appeared vaguely, with only a hiccup as a symptom.

## Introduction

In 1950, Peters and colleagues coined the term "Cerebral salt wasting syndrome" (CSWS) to describe how cerebral disorders could impair the kidneys' ability to conserve salt, resulting in salt depletion and extracellular fluid loss. Although other reports supported this phenomenon, it was overshadowed by the discovery of the inappropriate antidiuretic hormone secretion (SIADH) syndrome in 1957. However, evidence suggests that many patients with intracranial disease experience CSWS as initially described. This occurrence has significant clinical implications because the accepted treatment for SIADH, salt restriction, is the opposite of the accepted treatment for hyponatremia caused by CSWS, which is salt and water supplementation. It may not be easy to distinguish between SIADH and CSWS in a clinical setting. The most important criterion is the volemic status [1]. 

In a study of 224 patients with isolated craniocerebral war injuries, 39 patients had polyuria with significant sodium disturbance, 21 of whom had hyponatremia and polyuria. In these polyureic patients, the development of hyponatremia raises the possibility of CSWS [2]. Some experts believe that CSWS causes hyponatremia as frequently as SIADH, particularly in neurosurgical patients. Other research suggests that this syndrome accounts for no more than 6% of hyponatremia cases in patients with acute brain injuries.

Common symptoms of hyponatremia include nausea, vomiting, confusion, lethargy, coma, and even death. Another unconventional and infrequent symptom is hiccups, which have a solid and independent link with hyponatremia, particularly in hospitalized patients. The dose-response relationship demonstrated by a study conducted in India on 50 consecutive patients suggests a causal relationship [3]. A case report reported a hyponatremia-induced hiccup as an unusual COVID-19 symptom. A patient had a sodium level of 103 mEq/L. After correcting the hyponatremia, the hiccup stopped [4]. 

In our case report, we attempted to highlight hiccups as an unusual but striking manifestation of hyponatremia. Also, we tried to highlight CSWS as a potential cause of hyponatremia after ischemic stroke and how to approach it differently than SIADH, which is its closest differential.

## Case presentation

A 76-year-old male from Bangladesh suddenly started complaining of severe weakness and confusion along with hiccups, so he was taken to a nearby hospital. He had a medical history of hypertension (HTN) and type 2 diabetes mellitus (T2DM). Initially, he used to take amlodipine as an anti-hypertensive, which was later switched to thiazide diuretics as he was getting ankle edema as a side effect of amlodipine. He was compliant with his medication. On presentation, his blood pressure was 145/81 mmHg, his pulse was 81 beats per minute, and his oxygen saturation was 93%-94% on room air. A CT scan confirmed the ischemic stroke, and he was transferred to the intensive care unit. Thrombolytics were provided as per the protocol. At the time of admission, serum sodium concentration was 97 mEq/L (normal value: 135-145 mEq/L), serum osmolality was 215 mOsm/kg, urine osmolality was 655 mOsm/kg (normal value: 500-850 mOsm/kg), urine potassium concentration was 121 mEq/L (normal value: 25-125 mEq/L), and urine sodium concentration was 164 mEq/L (normal value: 40-220 mEq/L). Hyponatremia was thought to be caused by dehydration or medication use. He was treated with 3% sodium chloride to correct his sodium level back to a normal level and was discharged. One month later, he again developed weakness and bouts of intractable hiccups. He was brought back to the hospital, and his sodium level was found to be 120 mEq/L. There was no change in the medications in the meantime. Any other significant history or lifestyle modifications that could cause recurrent hyponatremia was not identified. After the correction of the hyponatremia, the hiccups were resolved. In this study, the potential causes of hyponatremia in his case were looked for. SIADH was kept as a differential because he had a history of pulmonary tuberculosis. The cerebral salt wasting syndrome was also considered because of a recent ischemic stroke. Other causes like hypothyroidism and adrenal insufficiency were ruled out based on normal adrenocorticotropic hormone (ACTH) and thyroid-stimulating hormone (TSH) levels. Urine and blood osmolality tests (Table 1) and urine electrolyte levels (Table 2) were ordered, and the results are charted in the following tables.

**Table 1:** Blood and urine osmolality test results

Test	Result	Reference Value
Blood Osmolality	265 mOsm/Kg	276.0-295.0 mOsm/kg
Urine Osmolality	239 mOsm/Kg	60-1400 mOsm/kg

**Table 2:** Urine electrolytes at 24 hours

Test	Result	Unit	Reference value
Urinary sodium	318.00	mmol/24 hrs	40-220
Urinary potassium	69.50	mmol/24 hrs	25-125
Urinary Chloride	370.00	mmol/24 hrs	110-250
Urine volume	6500	ml	

As CSWS is a type of hypotonic hyponatremia, we expect to find low serum osmolality, high urine output, and high urine sodium. The patient also had a recent history of ischemic stroke. Based on the patient history and lab results, the diagnosis of cerebral salt wasting syndrome was confirmed. He was prescribed a table salt supplement and pottasium syrup diluted in water. His symptoms are well controlled so far.

## Discussion

Hiccups are a common phenomenon characterized by the sudden, repetitive, and involuntary contraction of the diaphragm and other intercostal muscles. Based on the duration, the hiccup is classified into three sub-groups: acute attacks that last less than 48 hours; persistent hiccups, which last more than 48 hours but less than one month; and intractable hiccups, which last more than one month [5]. Although acute hiccup attacks are usually benign, persistent and intractable hiccups should be evaluated to rule out the underlying cause, as these may impact the quality of life by impairing sleep, eating habits, and social interaction. Rarely, hiccups can appear as a symptom of cerebrovascular disease-related hyponatremia. Among the various underlying causes, the three systems commonly associated with hiccups are neurological, gastrointestinal, and cardiovascular [6], as shown in Table 3.

**Table 3:** Underlying causes of persistent hiccups

System	Causes
Neurological	Cerebrovascular disease, Brain tumor Intracranial injury
Gastrointestinal	Reflux esophagitis, Large hiatal hernia
Cardiovascular	Myocardial infarction, Pericardial and aortic aneurysm
Others	Foreign body in the external auditory canal, Uremia Hyperglycemia

In our practice, hyponatremia is the most common electrolyte-disordered encounter. Cerebrovascular disease is sometimes associated with hyponatremia and may present as a hiccup. CSWS is a rare but potential cause of hyponatremia. It is characterized by hyponatremia, elevated urine sodium, and hypovolemia [7]. It can arise in the setting of multi-spectrum CNS pathologies, most notably due to aneurysmal subarachnoid bleeding [8]. In this case report, we attempted to demonstrate the correlation between stroke/cerebrovascular disease and the rare presentation of hiccups [9]. Our differential diagnoses for hyponatremia were central diabetes insipidus, SIADH, CSWS, adrenal insufficiency, and hypothyroidism. All other causes were carefully excluded.

**Figure 1:**
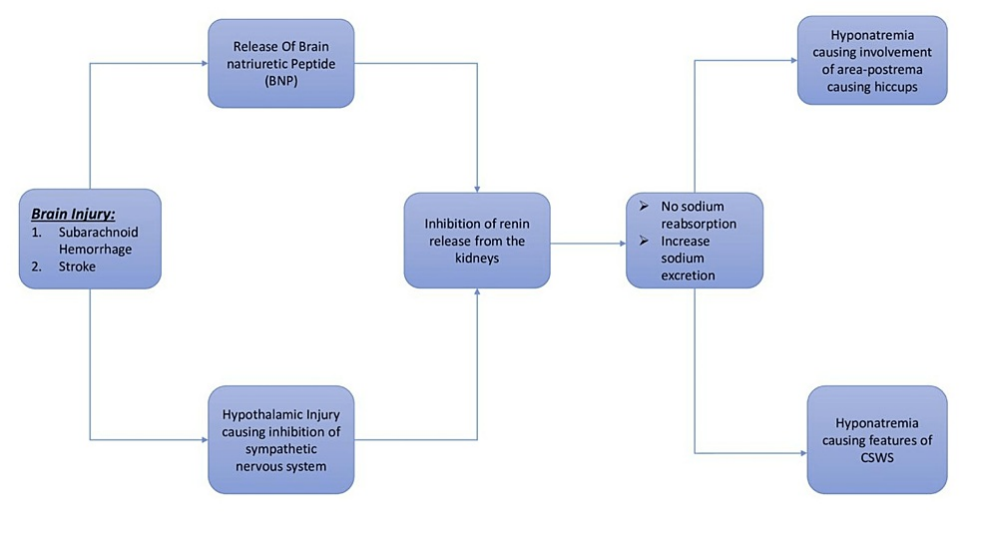
Two mechanisms behind the pathogenesis of the cerebral salt wasting syndrome (CSWS). Self-illustrated image

According to one theory, brain natriuretic peptide (BNP) is released after traumatic brain injury and acts on the collecting duct of the renal tubules, preventing sodium reabsorption and renin release. Another theory is that due to hypothalamic injury, the sympathetic nervous system no longer stimulates sodium reabsorption and renin release. These two theories are depicted below (Figure 1).

The most important differential diagnosis of CSWS is SIADH, as both present with hypotonic hyponatremia, urine sodium > 40 mEq/L, and hyperuricemia. However, their fundamental differences lie in volume status, urine output, and serum urea level. It is essential to differentiate CSWS from its close ally, i.e., SIADH, as the treatments are opposite [10] (Table 4).

**Table 4:** Differences between SIADH and CSWS CNS: Central nervous system; SSRI: Selective serotonin reuptake inhibitors; NSAIDs: Non-steroidal anti-inflammatory drugs; SIADH: Syndrome of inappropriate antidiuretic hormone secretion; CSWS: Cerebral salt wasting syndrome

Traits	SIADH	CSWS
Causes	CNS (Trauma, Stroke, Hemorrhage) Drugs (SSRI, Carbamazepine, NSAIDs) Ectopic (Small cell lung cancer) Nausea/ Pain	Subarachnoid Hemorrhage Stroke
Pathogenesis	Increase water reabsorption from renal tubules	Sodium loss from renal tubules
Volume status	Euvolemia	Hypovolemia
Urine volume	Decrease	Increase
Role of fluid in the treatment	Fluid restriction	Fluid supplementation
Urine Sodium	Normal	Increased

The calculation of fractional excretion of uric acid (FEUA) before and after hyponatremia correction has been proposed as a method for distinguishing SIADH from cerebral salt wasting. According to this theory, FEUA is greater than 11% in both SIADH and salt wasting before hyponatremia correction. An FEUA that remains >11 percent after correction of hyponatremia indicates salt wasting caused by impaired proximal tubule sodium reabsorption. In contrast, an FEUA of 11% identifies patients with SIADH. However, serial FEUA measurements have yet to be validated against a consistent, rigorous, and convincing gold standard for detecting salt wasting [10]. 

The main goals of treatment in CSWS are volume repletion and correction of hyponatremia. In mild to moderate cases, hyponatremia is corrected by isotonic saline. In severe cases, 3% hypertonic saline is used. In refractory cases, fludrocortisone can be considered [7].

## Conclusions

In our case report, we attempted to bring attention to hiccups as an uncommon symptom of hyponatremia. Hiccups are not a common symptom of hyponatremia, so that they could be overlooked. We have highlighted the differences in the diagnostic approach between cerebral salt wasting syndrome and inappropriate antidiuretic hormone release syndrome.
